# Four decades of HIV/AIDS—where do we stand?

**DOI:** 10.1016/j.eclinm.2021.100943

**Published:** 2021-05-27

The HIV/AIDS pandemic remains one of the world's biggest health challenges, particularly in low-income and middle-income countries. Since the first reported cases of AIDS in June, 1981, the disease has claimed the lives of almost 33 million people, and at the end of 2019, UNAIDS estimated that 38 million people were living with HIV. As we approach the 40th anniversary of the identification of the disease, we highlight key moments that defined the HIV/AIDS pandemic over the past four decades.

In the summer of 1981, the US Centers for Disease Control (CDC) documented cases of Pneumocystis carinii pneumonia and Kaposi's sarcoma, usually reported in immunosuppressed patients, in previously healthy young men. The number of cases continued to increase, with the majority reported in men who had sex with men and in people who injected drugs. Notably, the patients exhibited defects in cellular immunity and had a high mortality. In September, 1982, the CDC issued a case definition for the new disease, which they called acquired immune deficiency syndrome (AIDS). The disease was also observed in patients who had received transfusions of blood or blood products, sexual partners of patients with AIDS, and children born to mothers with the disease. Consequently, in March, 1983, the CDC suggested that AIDS might be caused by an infectious agent transmitted sexually, via blood products, or by vertical transmission. However, it would be another year until a retrovirus, later named human immunodeficiency virus or HIV, was confirmed as the cause of the disease.

The identification of HIV led to the development of the first commercial blood test for the detection of the virus, which was approved by the FDA in March, 1985. The test consisted of an enzyme-linked immunosorbent assay that detected antibodies against the virus in the blood and was an unprecedented scientific breakthrough at the time, as it was developed in only 9 months. The test was initially used to screen blood products in blood banks; however, by March, 1986, testing was extended to people in high-risk groups. Investigation of the viral replication cycle led to the identification of the first antiretroviral drug, zidovudine, which was licensed by the FDA in March, 1987. Zidovudine, a reverse transcriptase inhibitor, decreased the amount of HIV RNA in the blood, and produced a modest benefit in the life expectancy of patients. However, it was almost 10 years later, in 1996, that a three-drug regimen, known as highly active antiretroviral therapy, heralded a new era in the treatment of HIV, and suppressed the concentration of the virus to reduce disease progression and transmission. Another important advance in the fight against HIV was the introduction of pre-exposure prophylaxis (PrEP) for HIV prevention in 2012. Several randomised, placebo-controlled trials have shown that a once-daily PrEP regimen is highly effective in preventing HIV infection.

Currently over 20 approved medications exist for the treatment of HIV. Strict patient adherence to therapy is essential to ensure viral suppression; however, treatment complexity and side effects can lead to non-adherence. This issue, together with drug resistance, highlights the need for novel agents and delivery systems. In 2018, the FDA approved the use of the humanised monoclonal antibody ibalizumab for multidrug-resistant HIV, a first-in-class agent that binds the CD4 receptor on T cells, thereby preventing viral entry and preserving immune function. In late 2020, the European Medicines Agency approved the use of the first two injectable long-acting antiretroviral agents, rilpivirine and cabotegravir, which can be administered monthly or bi-monthly instead of daily tablets, and which are associated with higher treatment satisfaction than oral administration.

Although pharmaceutical advances are instrumental in the HIV/AIDS response, behavioural and social interventions are also necessary. Access to HIV testing, sex education, harm reduction programmes for people who injected drugs, and access to care for the most vulnerable groups are essential components of HIV prevention strategies. Equally crucial are education and awareness campaigns to improve public knowledge and decrease stigma. However, political and socioeconomic factors can result in ineffective preventive measures in the hardest hit areas, such as sub-Saharan Africa, which has the highest prevalence of HIV globally. World AIDS Day, first commemorated in 1988, is an official global health campaign endorsed by the WHO, and is dedicated to increasing public awareness. In 1996, the UNAIDS was established to lead a global response to prevent transmission of HIV and improve the care of those already living with the virus, in an effort to reduce the disease burden.

Despite these efforts, the global HIV/AIDS pandemic is far from over. The UNAIDS reported that approximately 1 · 7 million people became newly infected with HIV in 2019. Moreover, an UNAIDS report released in July, 2020, revealed that the global 90-90-90 HIV treatment targets (ie, 90% of people living with HIV know their HIV status, of whom 90% are receiving antiretroviral treatment and of whom 90% have viral suppression), set for 2020 were not reached, resulting in an additional 3 · 5 million new infections and 820 000 deaths. This set back is likely to be further exacerbated by the COVID-19 pandemic, as modelling data by Alexandra Hogan and colleagues from the Imperial College London (London, UK) published in The Lancet Global Health in September, 2020, showed that interruption to antiretroviral therapy as a result of the pandemic could increase HIV mortality by 10% within 5 years in high-burden settings.

Considering the remarkable scientific advances and global collaborations over the course of four decades, and with the promise of novel therapies such as the two ongoing trials of HIV vaccine candidates, ending AIDS as a public health threat by 2030, as ambitiously outlined in the UN Sustainable Development Goals, could be achievable. However, our biggest challenge is to ensure that strategies for HIV prevention and treatment are deployed in an equitable manner so that no one is left behind.

*EClinicalMedicine*

